# Food—Sleep—Profuse Perspiration

**Published:** 1862-02

**Authors:** 


CHICAGO
MEDICAL JOURNAL.

VOL. V.] FEBRUARY, 1862. [No. 2.

### SELECTED.

### FOOD—SLEEP—PROFUSE PERSPIRATION.

*From Dr. Ware's Lectures on General Therapeutics*

It will be seen, from the statements already made, that one of the most potent agencies in promoting recovery from chronic affections is a perfect performance of the function of nutrition; and that, with a view to this, a due regulation of the diet is required. The general principles by which we are to be guided in this regulation have been stated, but there still remain a few other considerations that are to be kept in view.

Every case in regard to its management in this respect is to be studied by itself, and we are not to be governed by any arbitrary rules founded upon the supposed requirements of particular diseases. As has been formerly stated, precisely the same disease as to its pathological character, may require the most opposite arrangements as to diet in different persons, as well as in many other particulars. In pulmonary consumption, for example, one patient will thrive best upon a milk and vegetable diet—another upon a diet of animal food and stimulants. Why, we know not; we are to be governed by the fact that it is so, and not by any preconceived opinion that such or such kinds of food are suited to what we conceive to be the nature of the disease. The same is true of all other affections. It is true that in beginning the treatment we must be guided by certain general notions as to the nature of the case and its relations to particular kinds of food; but in this way we can only approximate to a true judgment of what the constitution of the patient requires. It is only by considering each case as an experiment, to be judged of on its own merits, that we can arrive at a satisfactory conclusion in what way it is to be managed.

Quantity has an important relation to the perfection, not only of the primary digestion of food, but also to the ultimate adaptation of the results of digestion to a perfect nutrition. The kind of food may be suitable, but a small excess in quantity may seriously interfere with its conversion into a proper material for nutrition. This small excess is often felt in various ways during its presence in the digestive organs: by the state of the mouth, the breathing, the head, the *malaise*, and a general aggravation of symptoms; but even when not felt here or in addition to this, it may afterwards prejudice the progress of disease. The organs of assimilation can only perfectly prepare a certain quantity; any quantity beyond this is liable to introduce into the circulation, and present to the diseased parts, a crude and imperfectly elaborated material, which, although it may be borne with impunity by organs in a state of health, is liable to impair the sanatory process in those that are in a state of disease. In a state of health there is a power of selection which enables parts to correct in some measure the errors of the assimilation, to eliminate such portions of the material as are not suited to their purposes, and to cast them off by the excretions. In a state of disease, we have reason to believe, this power is impaired, or at least cannot be exercised without interfering with the attempt which is making to return to the normal condition. As a general rule, it is safer that the quantity should be somewhat less than can be satisfactorily assimilated, rather than greater. This is a frequent source of evil. Apparent weakness is so common a condition of those laboring under chronic disease, and is so obvious both to the patient and those about him, that there naturally arises an idea that this weakness is the principal evil to be contended with, and that its relief depends upon the amount of food that can be taken without positive and direct injury. In point of fact, the removal of weakness does not depend upon the quantity alone of nutritient introduced into the blood, but also upon its quality; and upon the exactness with which it is capable of being applied to the necessities of the organs.

The form in which food is presented and the mode of its preparation, are also important items in the management of diet. This includes the question of a liquid or a solid diet, and also the degree of its dilution. Some patients digest liquids better than solids; and, of the liquids, those that are not highly concentrated. This can only be determined by observation, because differences in these respects are very common among those whose condition appears the same. Under the head of preparation are included all the particulars of the cooking, the mixture and the seasoning of food. So wide a subject can only be superficially glanced at. For the most part we are to prefer simple food, with no great variety at each single meal, but with considerable variety at successive ones, so far as this is compatible with the character of the case. Food which when alone is very successfully managed by the digestive organs, may cease to be so when combined with others. Of this we have the strongest example in mixtures where by the process of cookery they become pervaded by some form of oil; and this is more especially the case where its chemical character is changed by heat; but the same takes place, in a less degree, with regard to sugar. Such combinations are withstood by many constitutions, sometimes permanently—by many only temporarily; but I am persuaded that the processes of cookery, of which these are samples only, are closely connected with that imperfect assimilation, and consequently that vicious nutrition, which not only hinder recovery from chronic diseases, but also have much to do with their origination. Mankind in general, and even physicians, are not sufficiently ready to look far back into the whole habits of life to account for disease. They attribute it to recent occurrences. But in the case of chronic affections at least, it is quite certain that, although their obvious onset may be sudden and determined by recent events, yet their existence is due to causes previously operating gradually and insensibly. Imperfect assimilation and faulty nutrition are probably the principal of these, and of other causes which contribute, such as cold, heat, bad air, the state of the mind, etc., it is probable that their efficiency is much due to their interference, either directly or indirectly, with the perfection of this fundamental function of life, either generally or locally. So also the distinct improvement in chronic disease from food, air and climate, from suitable exercise, from an improved condition of mind, and various other circumstances, is due to their influence on the digestion, the assimilation and the appropriation of the aliment.

The selection of a suitable diet is not, then, the only point of view in which this subject requires to be considered. The circumstances which are capable of influencing the whole digestive, assimilating and nutritive processes, are of equal importance. The common experience of life shows what a variety of causes are constantly modifying these fundamental operations. Affections of the mind change the appetite and digestion, and both the secretions and excretions, all of which are component parts of them. Sudden passion will deprave the character of the nurse's milk so as to produce sickness and vomiting in the child, as well as improper food. Grief will produce a bad taste in the mouth, a foul breath, a disturbed urine. Want of sleep will do the same, and will reduce the weight and the strength. Improper ventilation, foul air, poisonous exhalations, operate in an analogous way, also cold, heat, fatigue, &c., &c. Whatever, in fact, may be the primary effects of these various causes, their ultimate influence, so far as they are causes of disease, or preventives of recovery, depends upon their interference with the perfect performance of the great function of nutrition. In speaking, therefore, as we proceed, of the management of the patient as to many of its details, we are to understand that it is by their influence in this particular direction that their importance is chiefly to be measured.

In all forms and conditions of disease, both acute and chronic, the state of the patient as to sleep is an important consideration, both as regards his comfort, and also as regards the satisfactory progress of his case. The nature of this condition of animal life we do not fully understand; we only know that it is a necessary one and having a vast influence on the state of the system. Its purpose seems to be to afford an opportunity, by the suspension of certain activities of the system which require the exhaustion of those powers that emanate from the nervous system, for the reinforcement of those powers. It is also during sleep that the repair of the tissues by nutrition is provided for. Not that all nutrition is suspended during our waking hours, or that all waste is suspended during sleep, but that in the two states of sleeping and waking there is respectively a large predominance of the repair and the waste. Sleep is not merely rest, as it has been sometimes considered, an entire rest of all the organs at once. It is something specifically different. It is a condition of an entirely different nature, and a condition for which rest is not in any sense a substitute. The mere fact of existence, without exercise, without fatigue, the simple going on of life, implies a certain expenditure of force, which renders necessary at certain intervals a suspension of those functions of the brain and nervous system which are subservient to the phenomena of mind. It is possible that ordinary rest might afford an opportunity for the nutrition of all these tissues, except those which are the agents of the mind. But it seems to be necessary for the repair of these, that the functions of the mind should be also suspended. Of the physical condition of the brain in sleep, and also concerning the peculiar state of the mind in sleep, notwithstanding the many theories which have been formed concerning them, we know nothing with certainty, and this is not necessary to the practical management of the sick. What should guide us is the knowledge that a certain amount of sleep at proper intervals is an absolute necessity, and that its absence or its deficiency is always a great evil, and to be prevented by every passible means.

In acute diseases a sufficient amount of sleep is at once a favorable indication of the nature and issue of a case, and also is an important agent in the promotion of a favorable issue. Its absence, on the contrary, is, *pro tanto*, an unfavorable indication as to the issue, and also promotes an unfavorable issue. Want of sleep adds to the sufferings of the patient, and also to his exhaustion, and consequently interferes with the success of the sanitory process and impairs the power of recovery. In every point of view, then, the state of the patient in this respect becomes the object of special attention.

Salutary changes in the condition of a patient will be often found to take place during sleep, and to manifest themselves most obviously on awakening from that which has been sound and refreshing. But the sleep here is not so much the cause of the change as its consequence, and yet if any circumstance prevent the sleep, the favorable symptoms are less likely to occur. The general purpose in acute disease is, throughout, to discover those circumstances which may prevent sleep, and endeavor to counteract them. Thus, the absence of sleep may be caused by an irritable and loaded state of the bowels, and then anema may give relief; by the heat and irritation of the skin from a paroxysm of fever, and then cold sponging, the warm bath, a warm foot-bath with infusion of hops or poppies, may answer the purpose; or coldness of the feet may be the cause, and then warmth of any kind may be employed. Sleep should be procured without direct medication, where practicable, but this is not always practicable; and, when not, various articles can be given, which are sometimes successful. Such are all the ethereal medicines—tincture of hops and belladonna, and some others. But these very frequently fail, or succeed but for two or three nights. Where the evil is a very marked one, the experiment should be tried of procuring sleep by opiates. These, it is true, often fail, and often give occasion to a variety of uncomfortable feelings. How far these should influence us to abstain from their use, is one of the most delicate questions in practical medicine. As I shall have occasion to speak more particularly of the qualities of opium hereafter, in connection with some of its other uses, I shall only remark here that the procuring of sleep by it, is so important an object in many cases, that this beneficial effect is not to be sacrificed, where the necessity is urgent, without a fair trial of several preparations, and in more than one way. Where a full dose by the mouth either fails of effect, or produces unpleasant secondary effects, its administration by the rectum may succeed; and where full doses in both these ways fail, a repetition at short intervals through the day, of very small doses, such as five or six drops of laudanum, or its equivalent of some other preparation, will succeed. It must be confessed, however, that there are some patients so unfavorably affected by this drug, that in no way can it be endured.

In chronic diseases of all kinds, the same attention to procuring sufficient sleep is an important item in the treatment. In many cases where patients are afflicted with insomnolence as a prominent symptom, it will be found to depend upon some circumstance which is capable of removal. Thus, in some persons, taking any solid food after the middle of the day, or in others the entire omission of food, will prevent sleep. The remedy in such cases is sufficiently obvious. Where the sensation of hunger is the cause, the purpose is better answered by a meal of some very light digestible food, such as a cup of milk porridge, or of warm milk, than by anything more substantial and slow of digestion. Still to this, as to every rule of dietetics, there are many exceptions, but these are more likely to occur in persons who are not laboring under distinct disease, but are wakeful in their ordinary state of health.

The suggestions made with regard to acute diseases are, in a general may, applicable also to chronic. Opium in these is less likely to produce immediate ill effects, and it is also easier to counteract them; but, on the other hand, since in acute diseases the absence of sleep is a temporary evil, and one which subsides at convalescence, its use is less likely to degenerate into a habit. In chronic cases, therefore, so far as its ultimate effects are regarded, this consideration is of no small importance. In acute cases there is seldom any danger that the patient will be led to the continued use of opium after he gets well, and we may therefore avail ourselves of its beneficial effects without regard to the future; but in chronic ones this is far from being the case, and it will sometimes happen, where patients have had comfortable sleep procured for some time by this drug, that it is very difficult to wean them from its use, when their disease has subsided. In persons of feeble resolution and of nervous temperament, it may be as hard to cure them of the habit of depending upon opium, as of the use of tobacco or of alcoholic stimulants.

When, therefore, there is a prospect that opium will be likely to be required for a long time, every expedient should be first employed in order to answer the purpose, and a considerable amount of wakefulness be endured, rather than resort to it. Besides the articles above mentioned as applicable in acute cases, there are others more peculiarly adapted to chronic, such as hearing reading aloud, the recurrence of monotonous sounds, repeating from memory, going through arithmetical or other calculations, with the eyes shut. The state of skin may require alteration by warm bath; of the feet, when cold, by warm applications; of the head, when hot, by cold sponging. The mind may need tranquillizing. A disturbed state of the nerves may be present, which sundry articles have the reputation of soothing, and perhaps with reason, such as valerian, assafœtida, musk, castor and camphor. There are some milder popular remedies, such as warm infusions of sage, balm or catnip, a cup of wine whey, or of spirituous liquor and water. Something of the efficacy of these may properly be attributed to their effect upon the patient's mind. His expectation of effect from the remedy he has taken, and the direction of his attention to it, undoubtedly tends to render that effect more likely to occur. At any rate, sleep is sometimes thus procured, and occasionally by a succession of such expedients the object is secured for a long time, but more frequently they at length fail of any effect.

It sometimes happens that after a short nap on first going to bed, a person wakens without any known cause, and then remains obstinately watchful for many hours. In this case, if he rises, washes his face, hands and feet, walks about briskly for awhile and returns to bed, the charm may be broken and a continued sleep will ensue. Or he may rise and write or read with the same result. Too much or very little bed clothing in very susceptible persons, may prove a cause of wakefulness, even when the patient is not aware that he is too hot or too cold. The choice of measures must depend upon the condition of the patient, since it is obvious that many of those mentioned are not universally applicable. There is one injunction frequently insisted upon, viz: that drowsiness in the day should be resisted, lest it should prevent sleep at night. This is sometimes true, but not always. In a majority of wakeful persons, it has been rather found, that a nap in the afternoon does not prevent sound sleep at night, but rather promotes it. And this seems to be accounted for by the consideration, that the slumber in the day soothes and quiets the system, and removes that irritation of the nerves which is so likely to occur from various causes in the course of the day, and thus predispose to watchfulness in the night.

But in spite of every expedient, it must be confessed that this symptom is, in many chronic cases, an exceedingly difficult one to manage, and that many persons are at length obliged to rely upon some form of opium.

I have alluded above to the state of the skin in connection with the sleep. We may further speak of the state of this organ—a very important, as well as extensive one—as exercising no inconsiderable influence not only upon the comfort, but also the internal condition of the patient. A hot and dry state of the skin on the one hand, or a cold, contracted and shrunken one on the other, are always attended with discomfort, and should be remedied as far as practicable. It is to be acted upon as a general rule, to bring the skin as nearly as possible into its natural condition. Hence, a dry and hot skin should be kept moist and cool; a cold and very damp one, warm and dry; a contracted and shrunken one, active; by suitable applications. True it is only effects of disease which are thus removed, but the removal of effects may aid in the removal of causes—as correcting the very hot, dry skin, which attends pain in the head or fever, often diminishes both. An imitation of the natural soft state of the skin, where it is very hot and uncomfortable, by sponging with a mixture of glycerine and water, removes the irritation it occasions, and thus secondarily may reduce the pulse and respiration. I do not speak of these as direct remedies of disease, but as alleviants of symptoms, and thus aiding the progress of recovery according to its natural law.

We sometimes find the skin in a state which contributes not only to great discomfort, but seriously impedes recovery. It is soft, flabby, moist and pale. It has a sodden look and feel. The hands and fingers are corrugated as if they had been soaked in warm water. This is sometimes an effect of disease, but not unfrequently merely of mismanagement. In disease it occurs under various circumstances, as in hectic fever, acute rheumatism, inflammations within the abdomen—both mucous and serous—in many of the lesser puerperal affections, and in mammary abscess. Where not an essential product of disease, we find it has been produced by too warm and too close a room, and too much clothing to the person and the bed. Thus there are those, both among patients and nurses, whose chief apprehension in sickness is a nervous dread of cold. We find the sick person in bed, cased in all or more than all, the flannel and other appendages which are worn in health. If a woman, as is apt to be the case, she retains the woollen waistcoat, drawers and linen with which she got into bed when first seized; to this she has added a pair of woollen stockings and a thick petticoat. Thus equipped, the skin has become tender and sensitive. The least movement of the bed-clothes, or the slightest draught of air, produces a feeling of chilliness, and blanket and comforter, one after another, are heaped upon her; a muffler is pinned around her head, a shawl drawn around her shoulders, a jug of hot water is placed at her feet, the windows are hermetically sealed, and she is fed with hot teas. She still has frequent chills, and you find her hot, nervous and restless. Yet the essential symptoms may present nothing alarming. The pulse, though excited, may be very good, the tongue tolerably clean, the appetite not deficient, and no want of sleep except what may be fairly attributed to her surroundings, and these would rob the healthy of it. Here we have to regard the real tenderness of the skin, which has been thus created, and the nervous apprehension of the sufferer; but by removing all incumbrances from the person except a single loose cotten or flannel gown, by reducing gradually the burden of bed-clothes, by admitting cool fresh air into the room—avoiding its direct flow upon the person—by administering cooling drinks, and drying the skin thoroughly by sufficient frictions, the patient speedily passes from a state of comparative torment of body and mind, to one of ease and tranquillity.

Where this condition has not been artificially induced in a mild case, but accompanies a grave disease, the same general measures are adapted to mitigate it and give comfort, even although it necessarily continues. The cause and character of a permanently too copious sweating, is different in different cases. The sweat is sometimes sour, sometimes fœtid, sometimes highly saline, of a urinous odor or ammoniacal, or quite sticky and slimy. Various expedients for relief will suggest themselves, according to different circumstances, such as warm dry friction with a cotton or woollen towel, rubbing the skin with absorbent powders, such as powdered chalk, Indian meal or bran applied with a woollen mitten—washing with soap and water, with an alkaline solution, with a weak acid, with alcohol more or less diluted, and applied either warm or cold, according to the heat or any other possible indication, as the preference of the patient and its effects. Whenever articles are used which leave any residuum upon the skin, this is from time to time to be carefully sponged off. Merely bathing the whole body with olive oil, as in the sweats of phthisis, will be occasionally found to give some relief. Many articles internally administered have the reputation of diminishing these perspirations which are so debilitating to persons afflicted with exhausting chronic diseases, especially phthisis, scrofulous affections of the joints and chronic suppurations of all kinds. Among these, diluted sulphuric acid is most commonly employed, and most effectual ; but, besides this, acetate of lead, oxide of zinc, gallic acid, and other vegetable acids, are found more or less efficacious. Usually, however, the corrective influence is transient, and continues but a few days before the symptom returns. Sometimes the simple expedient of clothing the patient in a loose, light flannel nightgown is more successful and durable than anything else.—*Boston Medical Journal.*

